# Impact of Flood on Breastfeeding Practices at Flood Relief Camps of Pakistan

**DOI:** 10.12669/pjms.40.8.8488

**Published:** 2024-09

**Authors:** Wajiha Rizwan, Masood Sadiq, Mulazim Hussain Bukhari, Muqaddas Tasneem

**Affiliations:** 1 Wajiha Rizwan, MBBS, FCPS (Paediatric) Associate Professor, Pediatric Medicine Department University of Child Health Sciences/ The Children’s Hospital Lahore, Pakistan; 2 Masood Sadiq, MBBS, MRCP (UK), FRCP(Ed), FCPS (Paediatric Cardiology), FRCPCH (UK) Vice Chancellor University of Child Health Sciences/ The Children’s Hospital Lahore, Pakistan; 3 Mulazim Hussain Bukhari, MBBS, DCP, MPhil, PhD Principal, Azad Jammu Kashmir Medical College, Muzafrabad.; 4 Muqaddas Tasneem Head Nurse University of Child Health Sciences/ The Children’s Hospital Lahore, Pakistan

**Keywords:** Breastfeeding practices, Barriers, Flood, Pakistan, Developing country

## Abstract

**Objective:**

To explore impact of flood on breastfeeding practices and identify barriers in continuation of breastfeeding among mothers residing in flood relief camps.

**Methods:**

This exploratory observational study was conducted during visit of medical team of The University of Child Health Sciences, Children’s Hospital at flood relief camps of Sindh (7^th^ September to 12^th^ September, 2022) and south-west of Punjab province (18^th^ November to 20^th^ November, 2022). The data was collected on structured questionnaire from 40 lactating mothers residing in flood relief camps. Purposive sampling technique was used in this regard.

**Results:**

The mean age of breastfed children was 16.1±7.811 months. There was negative impact on breastfeeding practices (n=21, 52.5%) as frequency decreased in 18(45%) mothers and 3(7.5%) totally stopped breastfeeding. There was significant relation between pre-flood breastfeeding status and impact of flood on breastfeeding practices (p=0.001). The major barriers to appropriate breastfeeding were mother’s perception of insufficient breast milk due to inadequate diet (n=6, 15%) or depression and anxiety (n=4, 10%), mother’s illness (n=3, 7.5%), constant displacement (n=2, 5%) and provision of breast milk substitutes (n=2, 5%).

**Conclusion:**

There has been significant negative impact of flood on breastfeeding practices among lactating mothers residing in flood relief camps. Perception of decreased milk production due to inadequate diet and stress are major barriers in continuation of breastfeeding. Breastfeeding supportive services need to be integral component of flood crisis management.

## INTRODUCTION

The recent 2022 flooding in Pakistan is considered to be one of the major recent natural disasters as almost one-third of country was drowned, most likely as a consequence of climate change which is affecting many countries globally.^1^ Over 33 million Pakistanis were affected by these floods out of which 16 million were children. It caused displacement of almost 4.5 million people and over 1600 deaths.^2^ Pakistan is facing serious economic crisis due to destruction of agriculture and livestock leading to food shortage.^2^

Moreover, lack of clean drinking water, poor sanitation, outbreaks of water borne diseases like diarrhea, cholera and typhoid and loss of health care facilities has also created health crisis in the country. The children and pregnant females are considered to be highly vulnerable during any natural disaster. Globally every year, 760,000 children less than five years of age die during natural disasters mainly because of contaminated drinking water and waterborne infections.^2^-^4^ In Pakistan even before this crisis, one out of every nine children was malnourished, and that’s expected to worsen up further.^4^ In such circumstances continuation and promotion of breastfeeding can play a major role in improving health of infants and newborns particularly.^5^

Breastfeeding is considered to be the best and safest method of infant feeding during displacements and disaster. Breast milk is free of contamination and helps in building up immunity and prevents infections and malnutrition among infants.^6^ On the other hand the use of formula milk in the disaster relief camps has been associated with sudden increase in infant morbidity and mortality due to lack of clean water and proper equipment’s to prepare and store infant formulas.^5^ The available data gives some insight to the problems faced by displaced mothers in disaster relief camps (e.g., lack of support or privacy, unrestricted distribution of infant formula milks, maternal illness, misconceptions and myths about breast milk and feeding and gender-based constraint), it does not however, fully uncover barriers to mothers’ breastfeeding practices in flood relief camps in particular.^3^,^4^,^6^-^8^ Although such challenges related to breastfeeding in the time of humanitarian emergencies are encountered worldwide, they are much more problematic in low and middle-income countries like Pakistan.

There is very little data on impact of flood on breastfeeding practices among affected mothers and issues faced by them particularly in Pakistan.^4^ Given the vulnerability of nursing mothers and increasing child mortality rates in flood relief camps,^5^ we aimed to explore impact of flood on breastfeeding practices and identify barriers in continuation of breastfeeding among mothers having any child less than three years of age.

## METHODS

It was an exploratory study as due to limited data the prevalence of breastfeeding among flood relief camps of Pakistanis not known.^9^

The study was conducted on breastfeeding mothers in flood relief camps of one of the most affected areas of Sindh and south-west Punjab in Pakistan. The data was collected during visit of medical team of The University of Child Health Sciences, Children’s Hospital Lahore at Flood relief camps of Sehwan Sharif, Sindh (7th September to 12th September, 2022) and Dajal, south-west of Punjab (18th November to 20th November,2022). The sampling was non-probability purposive sampling and we gathered information regarding child age, gender, and breastfeeding status of mother before flood and displacement to flood relief camps and impact of flood on breastfeeding practices and major factor causing hindrance in continuation of exclusive breastfeeding.

### Ethical Approval:

The ethical approval was taken from the institutional review board of The Children’s Hospital & Institute of Child Health, Lahore (Ref. No. 2020-176-CHICH).

We collected data from 40 mothers who gave informed consent on pre-designed structured questionnaire based on previous literature.^3^,^4^,^7^,^8^-^10^ We included those mothers who were breastfeeding their < three year old child before flood and studied impact of flood on their breastfeeding routine. The mothers who never breastfed their child or left breastfeeding before flood for any other reason were excluded from study. A pilot study was conducted on six participants and then questionnaire modified by three senior pediatricians and one faculty member each from medical education department and preventive medicine. Fourteen participants were from Sindh and twenty-six from South Punjab.

The data was entered and analyzed using SPSS version 25. The numerical variables like age were presented as mean ± SD. The categorical variables like gender, breastfeeding status before and after flood was presented as frequency and percentages. The association between categorical variables was calculated through chi-square and p value ≤ 0.05 was considered significant.

## RESULTS

**Fig.1:**
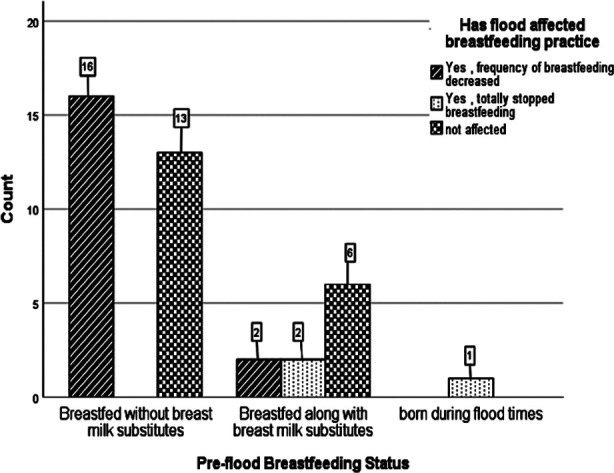
Relation of breastfeeding practices before flood times and impact of flood on it.

The mean age of the breastfed children was 16.1± 7.8 months (range 4 days to 30 months). Majority of mothers were illiterate (n=37, 92.5%). Male to female ratio of breastfed babies was 0.6:1 (Table-I). The gender of child was not found to be related to impact of flood on breastfeeding practices (p=0.912). The flood had significantly negative impact on breastfeeding practices among mothers in flood relief camp of South Punjab compared to Sindh (p= 0.003). There was significant relation between pre-flood breastfeeding status and impact of flood on breastfeeding practices (p=0.001) (Fig.1).

**Table-I:** Demographic data of study participants.

Variable	Number= n	Percentage%	P value
***Gender***			
Male	15	37.5%	0.980
Female	25	62.5%	
***Age***			
0-6 months	4	10%	0.104
>6 months	36	90%	
***Mother’s Education***			
Illiterate	37	92.5%	0.837
Below 5^th^ Standard	1	2.5%	
Completed high school	2	5%	
***Region***			
Sindh	14	35	0.003
South Punjab	26	65	
***Breastfeeding status Before Flood***	29	72.5%	
Breastfed without breast milk substitutes	10	25%	0.001
Breastfeeding along with breast milk substitutes	1	2.5%	
Born during Flood times			
***Breastfeeding status After Flood***			
Breastfed without breast milk substitutes	29	72.5%	
Breastfed along with breast milk substitutes	8	20%	
No breastfeeding, only breast milk substitutes	3	7.5%	
***Impact of Flood on Breastfeeding***			
Not affected	19	47.5%	
Yes, frequency decreased	18	45%	
Yes, totally Stopped Breastfeeding	3	7.5%	

**Fig.2:**
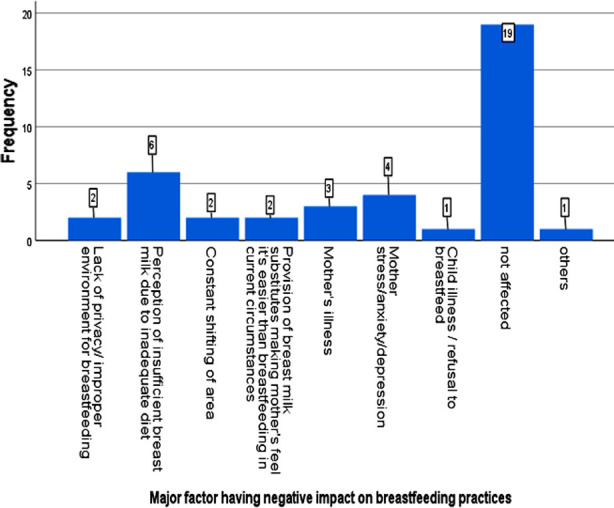
Factors having negative impact on breastfeeding practices.

**Fig.3:**
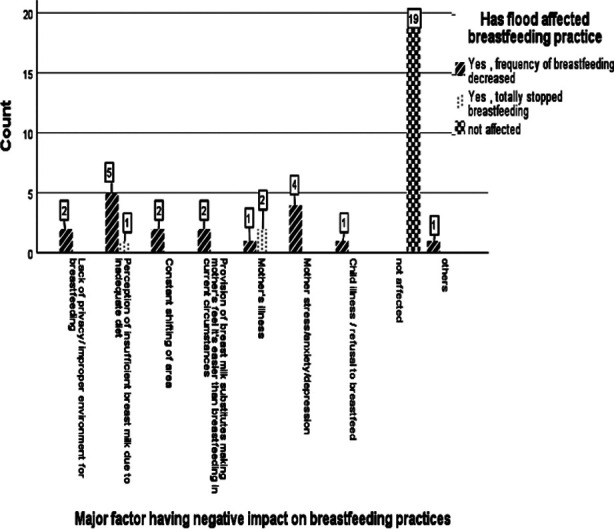
Impact of various factors on breastfeeding practices.

There was negative impact on breastfeeding practices (n=21, 52.5%) mothers in our study where 3 (7.5%) mothers totally stopped breastfeeding their babies. The major factor negatively affecting breastfeeding were mother’s perception of insufficient breast milk due to inadequate diet (n=6, 15%) or depression and anxiety (n=4, 10%), mother’s illness (n=3, 7.5%), constant displacement (n=2, 5%) and provision of breast milk substitutes (n=2, 5%). (Fig.2) The major factor that compelled mothers to completely hold breastfeeding was mother’s illness (n= 2, 5%). (Fig.3) In Sindh flood relief camp, all 3 (7.5%) mothers in whom breastfeeding was affected negatively had perception of insufficient breast milk production. Other factors were major reason of decreased breastfeeding practices in flood relief camp of Punjab.

## DISCUSSION

There was negative impact of flood on breastfeeding practices in over 50% of the mothers in our study where 7.5% mothers totally stopped breastfeeding their babies. Breastfeeding support services are highly crucial to prevent morbidity and mortality of infants during any natural disaster. Breast milk requires no preparation, has appropriate temperature and remains safe source of milk in environments with poor sanitation and unsafe drinking water.^9^ The challenges related to continuation of breastfeeding during flooding and subsequent displacements are prevalent worldwide, they are much more problematic in developing country like Pakistan where infant and child mortality rates are already high.^8^-^10^

The breast milk not only serves as food but potent medicine especially helpful in humanitarian crisis like flood.^11^The major factors negatively affecting breastfeeding was mother’s perception of insufficient breast milk due to inadequate diet and stress. This has previously been reported as well that during humanitarian emergencies, when mothers encounter food insecurity and stress, they believe that they unable to produce sufficient good quality breast milk. 4,9,10,12

Although mild to moderate maternal malnutrition or stress do not significantly alter the quantity or quality of their breast milk, many mothers quit breastfeeding or add on breast milk substitutes during natural disasters because of misconceptions about breastfeeding self-efficacy.^4^,^12^ Of three mothers (7.5%) who completely stopped breastfeeding, two (5%) did so due to their illness. The previous studies show that lactating mothers in relief camps stop breastfeeding mainly because of lack of privacy, stress or unhygienic conditions.^13^-^15^ There was significant relationship observed between pre-flood breastfeeding status and impact of flood on breastfeeding practices. It was also reported by Hirani SAA et al, that internally displaced mothers from Chitral shared that pre-flood cultural norm of breastfeeding encourages them to continue their breastfeeding practices despite various breastfeeding barriers.^9^

Though gender inequality surrounding child-feeding practices exists in South-Asian countries including Pakistan, our study showed no such disparity in breastfeeding practices in flood relief camps. Similar observation was reported by Hirani SAA et al., in flood relief camps of district Chitral in Khyber Pakhtunkhwa province of Pakistan.^9^

Among our study participant, a four days old boy born during flood times was not breastfed as mother believed that her milk production has ceased due to her poor food intake, hence she started giving infant formula milk to her baby. This is of major concern as around 70, 000 pregnant women in flood affected areas were expected to deliver in September, 2022.^16^ In such areas ravaged by recent flood, clean drinking water is unavailable that will make use of infant formula milk further harmful compared to breastfeeding.

In Punjab flood relief camp, negative impact of flood on breastfeeding practices was significantly more than seen in Sindh, one reason might be that we visited Sindh soon after flood crisis, whereas went to Rajanpur district of Punjab two months after flooding. Therefore, other factors like maternal stress, constant shifting or various maternal and child illness might have caused more harm over the time.

Currently, in Pakistan there is no policy or standardized program to support breastfeeding in situations of humanitarian crisis like flooding. Providing psychological support to lactating mothers to improve their breastfeeding self-efficacy, improving their physical and mental health and encouraging them to continue breastfeeding by highlighting its significance is the need of hour.

Our study findings give insight to the negative impact of floods on breastfeeding practices and can help to develop context-specific supportive strategies to improve breastfeeding practices in flood relief camps and hence, potentially decrease morbidity and mortality of children in Pakistan.

### Limitations:

The study utilized a relatively small sample size of 40 mothers from specific flood relief camps in Sindh and Punjab. Though this included all mothers’ full filling inclusion and exclusion criteria and giving informed consent. Moreover, the study provided a snapshot of breastfeeding practices during the immediate aftermath of the flood. However, a longitudinal analysis tracking changes in breastfeeding practices over time would offer a more comprehensive understanding of the situation.

## CONCLUSION

There has been a significant negative impact of flood on breastfeeding practices among lactating mothers residing in flood relief camps. Perception of decreased milk production due to inadequate diet and stress are major barriers in continuation of breastfeeding. Breastfeeding supportive services need to be integral component of flood crisis management. The proper understanding of these barriers will help policy makers and other stakeholders to improve and encourage breastfeeding practices in flood relief camps by developing context-specific interventions and decrease morbidity and mortality among young children.

### Authors’ Contribution:

**WR:** Drafting the article, data interpretation.

**MS:** Conceptualization and design, Critical review.

**MHB:** Data analysis, also responsible for the integrity and accuracy of the manuscript.

**MT:** Data collection and analysis.
